# Research progress of mitophagy and its targeted therapeutic strategies in rheumatoid arthritis

**DOI:** 10.3389/fimmu.2026.1959419

**Published:** 2026-10-15

**Authors:** Yuanyuan Zhang, Fengmei Ge, Xuebin Wang

**Affiliations:** Shandong Medical and Pharmaceutical University Hospital, Binzhou, China

**Keywords:** immune cells, inflammation, mitophagy, rheumatoid arthritis (RA), targeted therapy

## Abstract

Rheumatoid arthritis (RA) is a chronic, systemic autoimmune inflammatory disease characterized by persistent joint inflammation and progressive bone destruction. In recent years, mitophagy—a selective form of autophagy—has garnered increasing attention. As a critical cellular process, mitophagy specifically removes damaged mitochondria and maintains mitochondrial homeostasis and energy metabolic balance. Accumulating evidence suggests that mitophagy not only regulates cell survival and metabolic reprogramming but also plays a central role in preserving immune homeostasis, modulating immune cell polarization, and suppressing inflammatory signaling. This article reviews the molecular mechanisms of mitophagy and its pivotal role in RA, along with recent advances in mitophagy-targeted therapeutic strategies, aiming to provide novel insights for RA treatment.

## 1 Introduction

RA is a systemic autoimmune disorder characterized by synovial hyperplasia, inflammatory cell infiltration, pannus formation, and progressive cartilage and bone destruction, ultimately leading to joint deformity and functional impairment (1, 2). Although multiple treatment options are currently available, a subset of patients exhibits inadequate response to conventional therapies. Thus, identifying new therapeutic targets remains a key focus of RA research. Mitophagy, a selective form of autophagy responsible for the clearance of damaged or dysfunctional mitochondria (3), has emerged as a critical pathway. Studies indicate that mitophagy is impaired in RA patients, particularly in synovial fibroblasts and immune cells (4). Recent findings highlight mitochondrial dysfunction as a central contributor to RA pathogenesis (5). Synovial fibroblasts and immune cells in RA exhibit defective oxidative phosphorylation, excessive reactive oxygen species (ROS) production, impaired mitophagic activity, and metabolic reprogramming abnormalities (6, 7). These mitochondrial disturbances promote chronic inflammation, apoptosis resistance, and joint damage, potentially underpinning treatment resistance (8). The holistic approach of traditional Chinese medicine aligns with the multifactorial nature of RA and offers advantages in mitigating adverse effects associated with long-term immunosuppression. This review summarizes the immune-regulatory mechanisms of mitophagy in RA, discusses recent progress in targeting mitophagy for therapeutic intervention, and highlights the potential of traditional Chinese medicine and natural products in modulating mitophagy-related pathways, thereby bridging traditional medical concepts with modern molecular insights.

## 2 Molecular mechanism of mitophagy

Mitophagy enables cells to respond to external stressors and maintain mitochondrial quality and function by selectively eliminating redundant or dysfunctional mitochondria, thereby preserving cellular homeostasis (9). The process involves four key steps: first, damaged mitochondria undergo depolarization and loss of membrane potential, which serves as a trigger for mitophagy initiation. Second, the impaired mitochondria are engulfed by autophagosomes to form mitophagosomes. Third, the mitophagosome fuses with a lysosome, delivering the mitochondrial cargo for degradation. Finally, lysosomal acidic hydrolases degrade the mitochondrial contents, allowing the cell to recycle the resulting biomolecules (10, 11).

Three distinct forms of autophagy have been described: macroautophagy, microautophagy, and chaperone-mediated autophagy(CMA) (12). As a selective form of autophagy, mitophagy primarily relies on the macroautophagic machinery to specifically degrade damaged or superfluous mitochondria.Among these, macroautophagy is the most extensively studied. In this pathway, cells encapsulate intracellular substances by forming double-membrane autophagosomes that subsequently fuse with lysosomes to form autophagolysosomes, where the contents are degraded by lysosomal proteases. Microautophagy is a degradation process in eukaryotes in which cytoplasmic components are directly engulfed by lysosomes or through invagination of the vacuolar membrane, independent of autophagosome formation (13). Microautophagy is more selective than macroautophagy and can be initiated by signal molecules exposed on the surface of damaged organelles such as mitochondria or peroxisomes, leading to targeted lysosomal fusion. The resulting autophagic processes are named according to the targeted organelles; for example, mitophagy refers to the selective degradation of mitochondria, while pexophagy targets peroxisomes. Selective autophagy denotes such cargo-specific degradation. In contrast, CMA is a mechanistically distinct form of selective autophagy that degrades individual cytosolic proteins rather than whole organelles. CMA substrates are cytosolic proteins bearing a pentapeptide motif (related to KFERQ) in their amino acid sequence; they interact with cytoplasmic chaperones, which facilitates their targeting to lysosomes, where the substrate protein binds to lysosome-associated membrane protein 2A (LAMP-2A) and is translocated into the lysosome for degradation (14). Notably, because CMA degrades soluble cytosolic proteins rather than whole organelles, it does not directly participate in the degradation of whole mitochondria, which is executed by mitophagy.

Mitophagy mechanisms can be broadly classified into two categories: ubiquitin-dependent and non-ubiquitin-dependent pathways (15) (**Figure 1**). The ubiquitin-dependent pathway involves the ubiquitination of mitochondrial outer membrane proteins as a key step in autophagic recognition (16). The best-characterized pathway is the PINK1/Parkin pathway, which plays a central role in clearing damaged mitochondria through coordinated regulation of mitochondrial dynamics, translocation, and autophagy initiation (17). PTEN-induced kina se 1 (PINK1), encoded by the PINK 1 gene, is a serine/threonine kinase composed of 581 amino acid residues (18). Parkin functions cooperatively with ubiquitin - activating enzymes (E1) and ubiquitin - conjugating enzymes (E2) to covalently attach ubiquitin to substrate proteins, thereby mediating complete ubiquitination (19). In the canonical PINK1/Parkin pathway, mitochondrial damage leads to a loss of mitochondrial membrane potential (MMP, ΔΨm), impairing PINK1 import into the inner mitochondrial membrane (IMM). Consequently, PINK1 accumulates on the outer mitochondrial membrane (OMM), where it associates with the translocase of the outer membrane (TOM) complex and becomes stabilized. Activated PINK1 phosphorylates ubiquitin and recruits Parkin from the cytosol, inducing conformational changes that activate Parkin’s E3 ubiquitin ligase activity, thereby promoting ubiquitination of OMM proteins (20). Subsequently, the adaptor protein SQSTM1/p62 binds to both ubiquitinated mitochondrial proteins and microtubule-associated protein 1 light chain 3 (LC3), bridging ubiquitinated mitochondria to the autophagosomal membrane and enabling fusion with lysosomes to form autolysosomes, ultimately resulting in mitochondrial degradation. Accumulating evidence indicates that PINK1 and Parkin function in the same genetic pathway, with Parkin acting downstream of PINK1, and their interaction is essential for maintaining mitochondrial quality control (21). In addition to the classical PINK1/Parkin axis, Parkin-independent ubiquitin-mediated mitophagy pathways have been described (22); for example, mitochondrial-resident E3 ligase Mitochondrial E3 ubiquitin protein ligase 1(MUL1)-mediated stabilization of PINK1 can induce Parkin-independent mitophagy (23). In these pathways, PINK1-generated phospho-ubiquitin on the OMM directly recruits ubiquitin-binding autophagy receptors such as Optineurin(OPTN) and Nuclear dot protein 52 kDa(NDP52), which recognize ubiquitinated mitochondria and interact with LC3 to initiate mitophagy (22). Notably, Parkin-independent mitophagy is not synonymous with ubiquitin-independent mitophagy: the latter is mediated by mitochondrial membrane receptors such as NIX, BNIP3, and FUNDC1, which bind LC3 directly without requiring ubiquitination.

**Figure 1:**
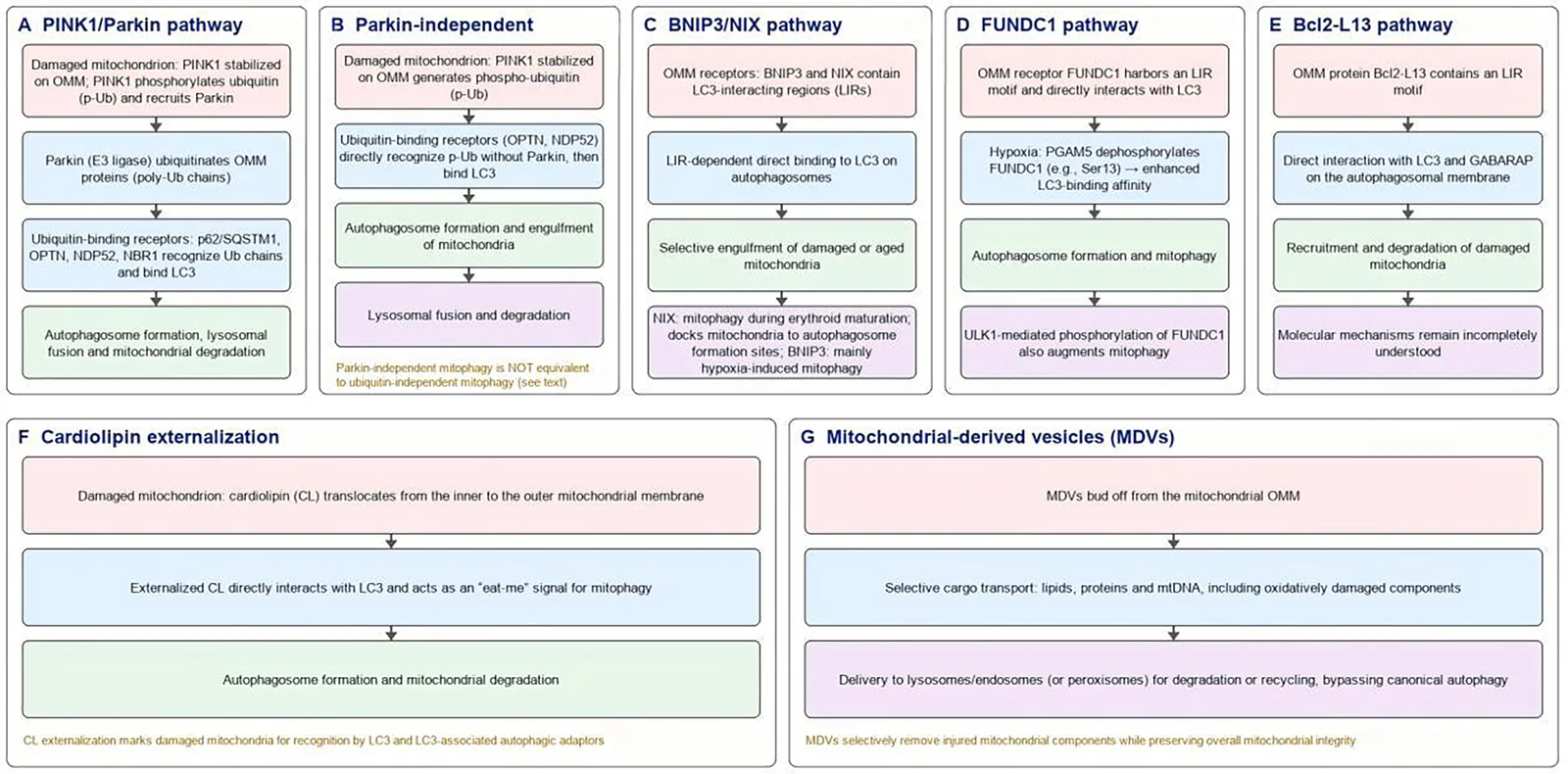
Mitophagy ubiquitin-dependent pathways and non-ubiquitin-dependent pathways. **(A)** The PINK1/Parkin pathway: upon mitochondrial depolarization, PINK1 is stabilized on the outer mitochondrial membrane (OMM) and phosphorylates ubiquitin, recruiting the E3 ubiquitin ligase Parkin; Parkin ubiquitinates OMM proteins, and ubiquitin-binding autophagy receptors (e.g., p62/SQSTM1, OPTN, NDP52) bridge ubiquitinated mitochondria to LC3-decorated autophagosomes, culminating in autophagosomal engulfment, lysosomal fusion, and mitochondrial degradation. **(B)** Parkin-independent, ubiquitin-dependent mitophagy: PINK1-generated phospho-ubiquitin directly recruits ubiquitin-binding receptors (OPTN, NDP52) in the absence of Parkin. **(C)** BNIP3/NIX pathway: the OMM receptors BNIP3 and NIX directly bind LC3 via LC3-interacting regions (LIRs), mediating selective mitochondrial engulfment. **(D)** FUNDC1 pathway: FUNDC1 interacts with LC3 through its LIR motif; hypoxia-induced dephosphorylation (e.g., PGAM5-mediated) and ULK1-mediated phosphorylation regulate its pro-mitophagic activity. **(E)** BCL2-L13 pathway: BCL2-L13 directly interacts with LC3 and GABARAP to recruit damaged mitochondria for degradation. **(F)** Cardiolipin externalization: upon mitochondrial damage, cardiolipin translocates from the inner mitochondrial membrane (IMM) to the OMM and serves as an ‘eat-me’ signal recognized by LC3. **(G)** Mitochondrial-derived vesicles (MDVs): small vesicles budding from the OMM transport selected mitochondrial components (lipids, proteins, mtDNA) to lysosomes or endosomes for degradation or recycling, bypassing canonical autophagy. Note that Parkin-independent mitophagy **(B)** is not equivalent to ubiquitin-independent, receptor-mediated mitophagy **(C-E)**.

In contrast, non-ubiquitin-dependent pathways involve mitochondrial receptors that directly mediate mitophagy without requiring ubiquitination. Receptor-mediated mitophagy selectively eliminates mitochondria through direct interactions between mitophagy receptors and components of the autophagic machinery, playing a pivotal role in mitochondrial quality control. To date, several receptor-mediated mitophagy pathways have been identified in mammals, including BCL2-interacting protein 3-like (BNIP3L/NIX), BNIP3, FUN14 domain containing 1 (FUNDC1), and BCL2-like 13 (Bcl2-L-13), among others (24).

These receptors function as autophagy adaptors by directly binding to LC3 without undergoing ubiquitination, thereby mediating selective mitophagy (25).

The BNIP3/NIX pathway represents a well-characterized receptor-mediated mitophagy mechanism. Both BNIP3 and NIX are outer mitochondrial membrane (OMM) proteins containing LC3-interacting regions (LIRs) (26). Through their LIR motifs, BNIP 3 and NIX directly interact with LC3 on autophagosome s, facilitating the selective engulfment of damaged or aged mitochondria (27). The role of NIX in mitochondrial clearance during erythrocyte maturation is well established, whereas BNIP3 primarily contributes to hypoxia- induced mitophagy (28). Recent reconstitution and structural studies have refined this model, demonstrating that transmembrane mitophagy receptors-including BNIP3, NIX, FUNDC1 and BCL2L13-engage the FAK family kinase-interacting protein of 200 kDa/ULK1 initiation complex and that BNIP3/NIX activity is subject to tight spatiotemporal and proteasomal quality control (29).

The FUNDC1 pathway is another well-defined receptor-mediated mitophagy mechanism. FUNDC1 is an OMM protein that harbors an LIR motif, allowing direct interaction with LC3(29). Under hypoxic conditions, dephosphorylation of specific residues in FUNDC1 enhances its binding affinity for LC3, thus promoting mitophagy (30). Additionally, phosphorylation of FUNDC1 by Unc-51 like autophagy activating kinase 1 (ULK1) can further augment its pro-mitophagic activity.

The Bcl2-L-13 pathway is a less extensively studied mechanism within receptor- mediated mitophagy. Bcl2-L-13 is an OMM protein containing an LIR motif, enabling direct interactions with LC3 and Gamma-aminobutyric acid receptor-associated protein(GABARAP) proteins on the autophagosomal membrane (31). This protein facilitates the degradation of depolarized mitochondria; however, the precise molecular mechanisms underlying this process remain incompletely understood.

In addition to the well-documented PINK1/Parkin pathway and receptor-mediated mitophagy pathways, several other mechanisms contribute to mitophagy. Two notable examples are cardiolipin externalization and the formation of mitochondrial-derived vesicles (MDVs). Cardiolipin, a phospholipid predominantly localized to the inner mitochondrial membrane (IMM), plays a critical role in maintaining mitochondrial structure and function (32). Upon mitochondrial damage, cardiolipin translocates to the OMM and interacts with LC3, initiating mitophagy. This process is mediated by NIX, which recruits autophagosomes to mitochondria (28). MDVs also participate in regulating mitophagy. These small vesicles bud off from the mitochondrial OMM and can transport various mitochondrial components — including lipids, proteins, and mitochondrial DNA (mtDNA) —to lysosomes or endosomes for degradation or recycling, thus bypassing canonical autophagy machinery (33). MDV formation appears to selectively target oxidatively damaged mitochondrial components, enabling removal of injured elements while preserving overall mitochondrial integrity (34).

## 3 Role of mitophagy in rheumatoid arthritis

### 3.1 mtDNA damage in RA

Mitochondrial dysfunction in rheumatoid arthritis (RA) is primarily driven by the hallmark features of the disease — namely, a chronic and highly inflammatory milieu. Inflammatory mediators can directly impair mitochondria, induce excessive reactive oxygen species (ROS) production, cause mtDNA damage, and disrupt mitophagy (35). Due to the absence of histone protection and its proximity to the electron transport chain (ETC), which is a major source of ROS, mtDNA is far more susceptible to oxidative stress than nuclear DNA. In the persistent inflammatory environment of RA, chronically elevated ROS levels can lead to mtDNA mutations and reduced mtDNA copy number (36). Given that mtDNA encodes essential subunits of the ETC, as well as transfer RNA (tRNA) and ribosomal RNA (rRNA), its impairment directly compromises ETC function, resulting in decreased adenosine triphosphate (ATP) production and further ROS leakage (37).

Moreover, damaged or extracellularly released mtDNA fragments can act as damage- associated molecular patterns (DAMPs), which are recognized by pattern recognition receptors on innate immune cells such as macrophages and neutrophils, thereby potently activating the innate immune response and intensifying the inflammatory response. This mechanism closely links mitochondrial damage to the pathological inflammation of RA. Studies have shown that the mutation frequency of mtDNA in inflamed synovial tissue is significantly increased and positively correlated with the degree of visible synovial inflammation, vascularity, and the levels of tumor necrosis factor α (TNF-α) and interferon-γ (IFN-γ) in synovial fluid (38).

Clinical studies have demonstrated that, compared with healthy controls, circulating cell-free DNA levels in the plasma of RA patients are markedly elevated, primarily due to a substantial increase in mtDNA. Quantitative analysis of mtDNA effectively distinguishes RA patients from healthy individuals with high sensitivity and specificity. Thus, quantitative assessment of plasma mtDNA holds significant potential for the early diagnosis of RA (39).

### 3.2 Mitophagy and cellular senescence

Mitophagy, as a central mechanism in maintaining mitochondrial homeostasis and regulating cellular aging, exerts a bidirectional influence on the immunopathological processes of RA. Functional imbalance in mitophagy can drive disease progression. Studies have revealed that CD4+ T cells in RA exhibit early-onset senescence features, including the senescence- associated secretory phenotype (SASP) and cytotoxic effector functions, which perpetuate inflammation and exacerbate disease progression (40, 41).

Mitophagy is significantly impaired in PD-1-expressing pathogenic senescent CD4+ T cells from RA patients. PD-1 signaling downregulates dynamin-related protein 1 (DRP1) expression by suppressing hypoxia-inducible factor 1-alpha (HIF-1 α) transcription, resulting in defective mitochondrial fission and impaired PINK1/Parkin-mediated mitophagy. This leads to abnormal accumulation of mitochondrial reactive oxygen species (mtROS) and ultimately promotes excessive SASP release, amplifying local joint inflammation and accelerating the progression of collagen-induced arthritis (CIA) in mice. Restoration of mitophagy can eliminate mtROS and suppress SASP, thereby mitigating inflammation and joint damage in RA (42), highlighting the critical role of mitochondrial fission and autophagy dysfunction in T-cell senescence and RA pathogenesis.

In the CD4+ T cell pro-aging pathway driven by heightened glucose metabolism, T cells from RA patients display abnormally enhanced mitophagy. A high-glucose environment promotes the accumulation of succinate, a tricarboxylic acid cycle intermediate, which induces succinylation of the transcription factor Zinc finger protein 76. This modification upregulates DRP1 expression and overactivates mitophagy, accelerating telomere shortening, cell cycle arrest, and senescence phenotypes in CD4+ T cells, thus promoting the onset and progression of RA. Inhibition of DRP1- dependent mitophagy can reverse premature T-cell aging and alleviate disease severity (43), providing crucial insights into the interplay between metabolic reprogramming and immune aging.

These findings indicate that impaired mitophagy exacerbates inflammation via the mtROS- SASP axis, whereas excessive autophagy contributes to pathological injury by accelerating T-cell senescence. Both pathways participate in immune dysregulation and joint destruction in RA. Furthermore, multiple T-cell subsets play pivotal roles in RA pathogenesis. Mitophagy significantly influences the differentiation of tissue-resident memory T (Trm) cells but does not affect type 1 helper T (Th1) or T helper 17 (Th17) cells. Notably, studies have shown that inhibition of mitophagy markedly reduces Trm cell numbers and attenuates the inflammatory response, evidenced by decreased levels of T-cell receptor β, IFN-γ, and interleukin-17A (IL-17A) in humanized RA chimeric models (44).

### 3.3 Mitophagy and bone metabolism

Studies have demonstrated that abnormal bone metabolism in RA is associated with activation of the mechanistic target of rapamycin complex 1 (mTORC1) signaling pathway, whereas chronic inflammation modulates the differentiation of pre-osteogenic cells via mitophagy. In preosteoblasts, tumor necrosis factor-alpha (TNF- α) has been reported to persistently activate the mTORC1 pathway, suppress Parkin-mediated mitophagy, induce mitochondrial damage, and impair osteogenic differentiation, thereby contributing to osteoporosis (45). In a separate model of RA synovial fibroblasts, TNF-alpha was instead shown to induce mitophagy, and inhibiting this mitophagy alleviated synovitis (46). Because these two findings derive from distinct cell types (preosteoblasts versus synovial fibroblasts), they should be interpreted separately rather than treated as interchangeable evidence. Rapamycin (RAPA), an mTORC1 inhibitor, enhances mitophagy and restores osteogenic differentiation by suppressing mTORC1 signaling and upregulating the PINK1-Parkin pathway, consequently promoting Parkin protein expression and activity. The combination of a TNF-alpha neutralizing antibody and RAPA has been shown to significantly ameliorate osteoporosis in antigen-induced arthritis (AIA) mouse models, suggesting a potential therapeutic strategy for RA-related osteoporosis (45).

### 3.4 Mitophagy and cartilage

In cartilage tissues from RA patients and collagen-induced arthritis (CIA) rats, acid-sensing ion channel 1a (ASIC1a) is markedly overexpressed, concomitant with excessive mitophagy and activation of PANoptosis(a unique programmed cell death pathway integrating pyroptosis, apoptosis, and necroptosis) (47). Mechanistically, ASIC1a upregulates calcineurin (CaN) expression, which competitively binds heat shock protein 70 (HSP70), disrupting the sirtuin 3 (SIRT3)- HSP70 complex. This disruption impairs mitochondrial translocation of SIRT3, resulting in defective mitochondrial deacetylation, reduced mitochondrial membrane potential, and accumulation of reactive oxygen species (ROS). Consequently, BNIP3/NIX-mediated, ubiquitin- independent excessive mitophagy is initiated, ultimately triggering PANoptosis in chondrocytes and accelerating cartilage degradation and bone erosion. *In vivo* studies confirm that silencing or inhibiting ASIC1a restores mitochondrial localization of SIRT3, suppresses excessive mitophagy and PANoptosis, and significantly alleviates joint damage. These findings reveal the ASIC1a – SIRT3 – excessive mitophagy – PANoptosis axis as a key molecular mechanism underlying cartilage destruction in RA, offering novel targets and therapeutic strategies for RA intervention through modulation of mitochondrial homeostasis.

A study on osteoarthritis revealed reduced expression levels of lysosome-associated membrane protein 1 (LAMP1) in chondrocytes, indicating a molecular association with mitochondrial dysfunction (48). Similarly, in newly diagnosed RA patients, decreased plasma protein levels of lysosome-associated membrane proteins (LAMPs) were detected, whereas LAMP gene expression in white blood cells showed no significant differences, suggesting lysosomal dysfunction at the protein level in RA (49). Additionally, autophagy contributes to T lymphocyte citrullination, promoting immune activation and chronic inflammation, which in turn leads to joint and cartilage damage. These alterations are linked to the resistance of RA synovial cells to apoptosis (50).

### 3.5 Mitophagy and fibroblast-like synoviocytes

In the hypoxic microenvironment characteristic of RA, hypoxia-inducible factor-1 α (HIF-1 α) transcriptionally co-activates both BNIP3-mediated mitophagy and NLRP3 inflammasome-mediated pyroptosis in fibroblast-like synoviocytes (FLS) (51). Notably, BNIP3-dependent mitophagy effectively attenuates hypoxia-induced pyroptosis of FLS and reduces the release of pro - inflammatory cytokines such as interleukin-1 β (IL-1 β) and interleukin-18 (IL-18) by clearing damaged mitochondria and lowering mitochondrial ROS (mtROS) levels, thus negatively regulating synovial inflammation. The HIF-1 α/BNIP3 pathway serves as a critical regulatory node balancing mitophagy and pyroptosis, and is implicated in the dysregulated survival and sustained inflammation of RA synovial cells.

PINK1/Parkin-mediated mitophagy is directly involved in the aberrant proliferation of fibroblast-like synoviocytes (RA-FLS) induced by hydrogen peroxide (H_2_O_2_) in rheumatoid arthritis.Oxidative stress triggered by H_2_O_2_ leads to mitochondrial dysfunction and elevated ROS levels in RA-FLS, which inhibit mitophagy by downregulating the expression of PINK1 and Parkin in mitochondria. This ultimately leads to abnormal cell proliferation, suppression of apoptosis, and promotion of synovial hyperplasia and disease progression in rheumatoid arthritis (RA). The ROS inhibitor N-acetylcysteine (NAC) can reverse the inhibitory effect of oxidative stress on the PINK1/Parkin pathway. Both the mitophagy activator FCCP and overexpression of PINK1/Parkin restore mitophagy levels, thereby suppressing RA-FLS proliferation and promoting their apoptosis. Adjuvant arthritis (AA) rat experiments further confirmed that NAC and FCCP significantly reduce arthritis scores, alleviate synovial hyperplasia, and promote synovial cell apoptosis (52).

Zhu et al. demonstrated that p62/SQSTM1 covalent oligomerization promotes the recruitment of Kelch-like ECH-associated protein 1(KEAP1) for autophagic degradation, thereby releasing nuclear factor-erythroid 2-related factor 2 (Nrf2) to activate downstream antioxidant and anti-inflammatory genes, which protects against oxidative and inflammatory joint destruction in RA *in vitro* and *in vivo (*53). Conversely, p62 knockdown reduced the antioxidant response and increased pro-inflammatory cytokine secretion upon TNF-α stimulation. Because p62 also functions as an autophagy receptor that links ubiquitinated cargo, including damaged mitochondria, to the autophagic machinery, this Nrf2-p62 axis may coordinate antioxidant defense with mitophagy to break the oxidative stress-inflammation vicious cycle in arthritis.

Liu et al. (54) revealed from an inflammatory perspective that angiopoietin-like 2 (ANGPTL2) deficiency impairs mitophagy by inhibiting insulin-like growth factor-binding protein 5 (IGFBP5), resulting in mitochondrial dysfunction in macrophages and persistent activation of the NLRP3 inflammasome, thereby exacerbating joint inflammation and bone erosion in collagen-induced arthritis (CIA) mice. These findings provide a novel theoretical basis for targeting mitophagy in RA therapy.

In addition, mitochondrial dynamics proteins of 49 kDa and 51 kDa (MiD49 and MiD51, collectively termed MiDs) are significantly upregulated in RA-FLS and patient serum, with expression levels positively correlated with clinical disease parameters (55). Knockdown of MiD49 or MiD51 alleviates CIA symptoms and suppresses RA-FLS invasiveness. Mechanistically, MiDs sustain aberrant FLS function by regulating PINK1-Parkin-mediated mitophagy and carnitine palmitoyltransferase 1A (CPT-1A)-dependent fatty acid β-oxidation. Inhibition of PINK1-Parkin reverses the invasive phenotype of RA-FLS. Therefore, MiD49/MiD51-mediated dysregulation of mitochondrial dynamics contributes to RA pathogenesis by modulating mitophagy and lipid metabolism, underscoring the critical role of mitochondrial dynamics proteins in metabolic reprogramming of synovial cells.

FUNDC1 is specifically downregulated in the synovial tissues of RA patients, and its expression level is significantly negatively correlated with joint injury and disease activity. Mechanistically, FUNDC1 deficiency directly causes impaired mitophagy, disrupted balance between mitochondrial fission and fusion, and collapse of mitochondrial quality control, leading to abnormal accumulation of mtROS. This further activates the mitogen-activated protein kinase (MAPK) signaling pathway, markedly enhancing migration and invasion capabilities of RA-FLS, and promoting secretion of matrix metalloproteinases and cathepsins. Ultimately, this exacerbates articular cartilage and bone destruction (56). Targeted restoration of FUNDC1-dependent mitophagy may offer a novel therapeutic strategy for controlling invasive inflammation and joint damage in RA.

Previous studies have demonstrated that, in contrast to the pro-inflammatory role of nuclear/cytoplasmic signal transducer and activator of transcription 3 (STAT3), mitochondrial STAT3 (mtSTAT3) represents a potential therapeutic target for rheumatoid arthritis (RA) (57, 58). ZnSO_4_, known as an inducer of mtSTAT3, has been shown to attenuate inflammation by improving mitochondrial function (59, 60). Lee et al. (61) demonstrated in collagen-induced arthritis (CIA) mice that mtSTAT3 overexpression significantly reduced arthritis scores, alleviated joint inflammation and osteochondral damage, and downregulated pro-inflammatory cytokines such as IL-17. Mechanistically, mtSTAT3 exerts protective effects in RA by restoring autophagic flux, suppressing reactive oxygen species (ROS) generation, and ameliorating IL-17-mediated autophagic dysfunction, highlighting a novel role for mitochondrial transcription factors in autoimmune regulation.

### 3.6 Mitophagy and macrophage polarization

Emerging evidence indicates that mitophagy modulates macrophage polarization in RA through regulation of mitochondrial function (62). For example, in THP-1 monocyte/macrophage cells (an *in vitro*, non-RA model), acrylamide was reported to induce PINK1-dependent mitophagy and promote an M2-like phenotype (63); importantly, this study did not directly demonstrate reduced inflammation in RA. In contrast, taurine has been reported to block PINK1-mediated mitophagy flux and the mitophagy-associated metabolic switch toward glycolysis, thereby suppressing M1 macrophage polarization (64). Of note, this taurine study was performed in cultured macrophages and was not conducted in an RA disease model. Together, these findings suggest that mitophagy regulates macrophage polarization in a context-dependent manner and that appropriately modulating mitophagy may suppress M1 polarization and mitigate the inflammatory response in RA, but direct evidence from RA-relevant systems remains limited (63).

The role of mitophagy in macrophage polarization is highly complex. Receptor-mediated mitophagy involving BNIP3L/NIX and FUNDC1 has been proposed to reduce mitochondrial mass and impair oxidative phosphorylation capacity by clearing damaged or dysfunctional mitochondria, thereby shifting macrophage metabolism toward glycolysis. However, evidence that this specific BNIP3L/NIX/FUNDC1-glycolysis axis directly supports M1 polarization remains indirect and is largely inferred from general autophagy and mitochondrial quality control studies (65). By contrast, low-fluoride exposure was reported to drive macrophage polarization through PINK1/Parkin-mediated mitochondrial autophagy in a non-RA setting (66), which supports a role for the PINK1/Parkin pathway rather than the BNIP3L/NIX/FUNDC1 axis in that particular response. Conversely, mitophagy acts as a mitochondrial quality control mechanism, preserving mitochondrial integrity and sustaining oxidative phosphorylation, which is essential for M2 macrophage metabolism (67). However, excessive mitophagy may disrupt cellular energy homeostasis and impair both M1 and M2 polarization. Given the close interplay between mitophagy and macrophage polarization, targeting this process may offer novel therapeutic strategies for RA.

Moreover, increased expression of triggering receptor expressed on myeloid cells 1 (TREM1) in the synovium has been identified as a key biomarker for assessing RA disease activity (68). Dai et al. (69) reported that TREM1 overexpression in macrophages significantly upregulated translocase of outer mitochondrial membrane 40 (TOMM40) and suppressed multiple mitophagy-related genes. Concurrently, E2F transcription factor 1 (E2F1), which contributes to macrophage metabolic reprogramming, inflammation, and bone destruction (70), was also upregulated upon TREM1 overexpression. Collectively, these findings indicate that TREM1 disrupts macrophage mitophagy via the E2F1-mediated transcriptional regulation of TOMM40, providing new insights into how inflammatory signaling pathways impair mitochondrial quality control.

Additionally, studies have revealed that the apelin receptor (APJ), a G protein-coupled receptor, plays a critical role in modulating the inflammatory and immune microenvironment. APJ activation enhances mitophagy through the AMP-activated protein kinase(AMPK)/BNIP3–PINK1–Parkin pathway, facilitates clearance of damaged mitochondria, reduces ROS accumulation, inhibits M1 macrophage polarization and NLRP3 inflammasome activation, thereby suppressing excessive osteoclast activation and ameliorating systemic inflammatory bone loss (71). APJ thus represents a promising therapeutic target for regulating the bone-immune microenvironment and treating inflammation- driven bone destruction.

It should be noted that mitophagy has often been inferred from a range of readouts across the studies above, including changes in LC3-II/p62 abundance, mitochondrial marker loss, and reporter systems. Because changes in general autophagy markers or in the steady-state abundance of mitophagy-related proteins alone do not necessarily reflect a bona fide increase in mitochondrial degradation (i.e., mitophagic flux), conclusions drawn from marker-based assays should be interpreted cautiously. Where possible, these should be complemented by flux measurements (for example, bafilomycin A1-blocked degradation, or mito-Keima/mt-mCherry-GFP reporter assays) and by cell-type-specific evidence (**Table 1**).

**Table 1:** Summary of experimental models, cell types, pathways, mitophagy readouts, and functional outcomes reported in RA-relevant studies.

Ref	Model/cell type	Pathway	Mitophagy readout	Functional outcome
(42)	CD4+PD-1+ T cells (RA patients); CIA mice	PINK1/Parkin (DRP1↓)	mt-Keima flux; PINK1/Parkin (WB); MtROS (MitoSOX)	SASP; joint inflammation
(43)	CD4+ T cells (RA patients); high glucose	DRP1/PINK1/Parkin	DRP1-dependent mitophagy markers; telomere shortening; cell-cycle arrest	T-cell senescence
(45)	Preosteoblasts	mTORC1–Parkin	Parkin expression; mitochondrial damage markers	Impaired osteogenesis; osteoporosis
(46)	RA synovial fibroblasts	TNF-α-induced mitophagy	PINK1/LC3-II (WB); PINK1–mitochondria colocalization (IF)	Mitophagy inhibition alleviates synovitis
(47)	Chondrocytes (RA/CIA)	BNIP3/NIX	LC3-EGFP-mCherry & mt-Keima flux; LC3B-II/p62, NIX/BNIP3 (WB); TEM	PANoptosis; cartilage/bone erosion
(51)	RA-FLS	HIF-1α/BNIP3	BNIP3-mediated mitophagy; NLRP3-mediated pyroptosis readouts (IL-1β, IL-18)	Reduced pyroptosis and cytokine release
(52)	RA-FLS (H_2_O_2_-treated)	PINK1/Parkin	LC3-II/I, p62, Beclin-1, PINK1/Parkin (WB); JC-1 ΔΨm; MitoSOX mtROS	FLS proliferation; synovial hyperplasia
(54)	Macrophages	ANGPTL2–IGFBP5	mito-Keima flux; PINK1/Parkin, LC3B-II, p62 (WB); LC3B–MitoTracker colocalization (IF)	NLRP3 activation; joint inflammation
(61)	CIA mice	mtSTAT3	LC3/p62, DRP1/Mfn2 (WB); LC3–LAMP1 colocalization (IF)	Reduced arthritis and bone damage
(63)	THP-1 cells (*in vitro*, non-RA)	PINK1	PINK1, LC3 (WB); LC3–MitoTracker colocalization (IF)	M2-like phenotype; no RA inflammation endpoint
(64)	Macrophages (*in vitro*, non-RA)	PINK1 (taurine)	PINK1/LC3B/p62 (WB); mitochondria–lysosome colocalization; BAF-A1-blocked flux	Suppresses M1 polarization
(69)	Macrophages	TREM1–E2F1–TOMM40	Mitophagy flux; TOMM40 and mitophagy-related gene expression; intracellular ROS	Inflammation; bone destruction
(72)	CIA mice	Src/FUNDC1	Src/FUNDC1-mediated mitophagy markers; macrophage polarization readouts	Macrophage polarization; improved RA
(73)	MH7A cells (CoCl_2_, *in vitro*)	HIF-1α/NLRP3	TEM (autophagosomes/mitophagosomes); LC3-II, p62 (WB); NLRP3/HIF-1α (WB/IF/ELISA)	Reduced pyroptosis *in vitro*
(74)	CIA mice	PINK1/Parkin (GSZG)	PINK1/Parkin-mediated mitophagy markers; ROS/NF-κB readouts	Suppressed osteoclastogenesis

## 4 Therapeutic strategies targeting mitophagy

Intervention studies on natural drugs and traditional Chinese medicine components targeting mitophagy are currently important research directions. Kaempferol is a naturally occurring flavonoid compound with multiple biological properties, such as anti-inflammatory and antioxidant activities (75). Li et al. (76) investigated the targeted regulatory effect of kaempferol on HIF signal- mediated mitophagy dysfunction from the perspective of synovial fibroblasts, revealing the potential application of flavonoids in ameliorating autophagy abnormalities induced by hypoxic microenvironments. However, this study has certain limitations, including a small sample size, lack of *in vivo* animal experiments, and the need for further investigation into the specific molecular targets of kaempferol.

In addition, melatonin is a multifunctional hormone with anti-inflammatory, antioxidant, and immunomodulatory properties (77). In collagen-induced arthritis (CIA) mice, melatonin promotes Src/FUNDC1-mediated mitophagy to regulate macrophage polarization, thereby alleviating rheumatoid arthritis (RA) (72). This study provides valuable insights for the development of novel mitophagy activators.

Du et al. (73) reported that, in a CoCl2-treated MH7A synovial fibroblast cell model *in vitro*, emodin modulates the balance between mitophagy and pyroptosis by inhibiting excessive mitophagy and suppresses the HIF-1alpha/NLRP3-mediated pyroptosis signaling pathway, reducing the release of IL-1beta and IL-18 and inhibiting synovial cell proliferation. These are *in vitro* findings and do not by themselves establish that emodin alleviates RA synovitis *in vivo*.

Guizhi-Shaoyao-Zhimu decoction (GSZD) is a traditional Chinese herbal formula with a long history of empirical use in the clinical management of RA. However, rigorous clinical evidence from controlled trials remains limited, and the evidence cited here comprises a corrected preclinical study (78) and collagen-induced arthritis (CIA) mouse experiments showing that Guizhi-Shaoyao-Zhimu granules enhance PINK1/Parkin-mediated mitophagy in osteoclast precursors, inhibit reactive oxygen species (ROS) production and the nuclear factor kappa-B (NF-κB) signaling, and suppress osteoclastogenesis (74). These findings should therefore be interpreted as preclinical rather than clinical proof of efficacy.

Collectively, these findings suggest that restoration of normal mitophagy function may represent a promising therapeutic strategy for RA, while also providing theoretical support for the modernization of traditional Chinese medicine. A 2025 systematic review further concluded that traditional Chinese medicine and natural products restore mitochondrial homeostasis in RA by enhancing mitophagy, reducing oxidative stress, and dampening inflammatory signaling, while emphasizing that pharmacokinetic standardization and biomarker-driven clinical trials are now required to advance these candidates (79).

Ultrasound (US)-activated piezoelectric catalytic therapy enables precise spatiotemporal induction of mitophagy, allowing targeted treatment of RA. Two-dimensional piezoelectric nanosheets (NSs) of Fe/BiOCl, engineered with US-responsive catalytic activity, efficiently generate electrons upon US stimulation. These reactive species selectively deplete protons (H^+^) on the outer mitochondrial membrane (OMM), disrupting the mitochondrial proton gradient and inhibiting ATP synthesis. The resultant mitochondrial membrane potential (MMP) depolarization triggers selective autophagy of damaged mitochondria in RA-affected cells, effectively suppressing the generation of mitochondrial reactive oxygen species (mtROS) and thereby ameliorating RA pathology (80).

Although natural compounds have demonstrated multi-target effects against rheumatoid arthritis (RA) and immunometabolic dysfunction at the mechanistic level, their therapeutic potential remains largely confined to academic research. Some studies have highlighted associated toxicological risks, such as potential hepatotoxicity or nephrotoxicity caused by certain herbal constituents, or the induction of immune system overactivation. The near absence of systematic safety evaluations — particularly for alkaloid compounds with inherent toxicity risks and engineered nanotherapies — represents a major barrier to clinical translation. Therefore, future preclinical development should prioritize toxicological assessment over efficacy evaluation, establish compound-specific risk assessment frameworks, and validate drug delivery systems to ensure long-term safety. Without these critical steps, natural compounds, despite their scientific promise, are unlikely to advance into clinical practice. The mitophagy-modulating candidates discussed above, together with their experimental models and evidence levels, are summarized in **Table 2**.

**Table 2:** Mitophagy-targeting therapeutic candidates reported in RA-relevant studies.

Agent/category	Target & pathway	Experimental model	Evidence level	Ref
Rapamycin	mTORC1-PINK1/Parkin activation	Preosteoblasts; AIA mice	*In vivo* animal	(45)
Kaempferol (flavonoid)	HIF-autophagy; MAPK8/NLRP3	RA fibroblast-like synoviocytes	*In vitro*	(75, 76)
Melatonin	Src/FUNDC1; macrophage polarization	CIA mice	*In vivo* animal	(72, 77)
Emodin (anthraquinone)	HIF-1alpha/NLRP3; mitophagy-pyroptosis balance	MH7A cells (CoCl2-treated)	*In vitro*	(73)
GSZG granules (TCM)	PINK1/Parkin in osteoclast precursors	CIA mice	*In vivo* animal	(74)
Fe/BiOCl nanosheets	US-activated *in situ* mitophagy	RA model	*In vivo* animal	(80)
Taurine (amino acid)	PINK1 mitophagy blockade	Cultured macrophages (non-RA)	*In vitro*	(64)

## 5 Conclusion and future prospects

Mitophagy plays a central regulatory role in the pathogenesis of RA, with its dysregulation implicated in immune activation, inflammatory amplification, and tissue damage. Aberrant mitophagy in synovial cells, macrophages, and T cells collectively contributes to joint inflammation and destruction. Imbalances in mitochondrial dynamics, dysfunction of receptor proteins, and disturbances in upstream signaling pathways constitute key mechanisms underlying impaired mitophagy. These findings underscore the significance of mitophagy in RA and provide novel insights for the precise classification and individualized management of the disease.

Therapeutic strategies targeting mitophagy, including natural products, traditional Chinese medicine (TCM) components, and ultrasound-based interventions, have shown promise in ameliorating disease symptoms and tissue damage in cell-culture systems and animal models (notably CIA, AIA, and AA models). It should be emphasized that the evidence summarized above is predominantly *in vitro* and preclinical; direct clinical evidence from RA patients remains limited. TCM has a long-standing history in RA management, characterized by notable efficacy and favorable safety profiles. Recent advances in extraction and development technologies for natural products have further enhanced the clinical applicability of TCM and other natural compounds. Nevertheless, prior to clinical implementation, comprehensive evaluation of their absorption, distribution, metabolism, excretion (ADME) properties, and potential organ toxicity is essential. Moreover, chemical synthesis and biosynthetic engineering may be required to enhance efficacy, optimize pharmacokinetics, and reduce adverse effects. Future research should integrate multi-omics approaches, such as metabolomics and network pharmacology, with organoid models to elucidate the complex, multi-component, multi-target, and multi-pathway mechanisms of action underlying TCM.

Future research should further elucidate the role of mitophagy in rheumatoid arthritis (RA) across multiple levels and accelerate the translation of basic findings into clinical applications. At the molecular level, single-cell multi-omics technologies should be employed to dissect the specific regulatory networks of mitophagy in distinct cell subpopulations, identify key regulatory nodes and disease-driving mechanisms, and investigate the crosstalk between mitophagy and other cellular processes such as ferroptosis and pyroptosis. In disease modeling, animal models that more accurately recapitulate human disease features should be developed to assess the differential efficacy of mitophagy interventions at various disease stages and to explore the synergistic mechanisms of combination therapies. In translational research, biomarkers based on mitochondrial functional assessment should be developed for disease diagnosis, monitoring of disease activity, and prediction of therapeutic response, along with standardized methodologies and clinical guidelines for mitophagy function testing. Regarding drug development, highly selective and safe mitophagy modulators should be screened, targeted delivery systems should be designed to enhance drug accumulation in affected tissues, and rigorous clinical trials should be conducted to evaluate therapeutic efficacy and safety. Additionally, the influence of individual variability on mitophagy function and treatment response should be considered, with exploration of precision medicine approaches tailored to genetic and phenotypic profiles. With ongoing advances in research methodologies and deeper mechanistic insights, mitophagy-targeted therapy is anticipated to become an integral component of the comprehensive management strategy for RA, offering improved clinical outcomes for patients.

## Glossary

RA: rheumatoid arthritis
ROS: reactive oxygen species
CMA: chaperone-mediated autophagy
LAMP-2A: Lysosome-associated membrane protein 2A
PINK1: PTEN-induced kinase 1
E1: ubiquitin-activating enzymes
E2: ubiquitin-conjugating enzymes
MMP: mitochondrial membrane p otential
IMM: inner mitochondrial membrane
TOM: translocase of the outer membrane
OMM: outer mitochondrial membrane
LC3: microtubule-associated protein 1 light chain 3
MUL1: Mitochondrial E3 ubiquitin protein ligase 1
OPTN: Optineurin
NDP52: Nuclear dot protein 52 kDa
BNIP3 L/NIX: BCL 2-interacting protein 3-like
FUNDC1: FUN14 domain containing 1
Bcl2-L-13: BCL2-like 13
LIRs: LC3-interacting regions
ULK1: Unc-51 Like Autophagy Activating Kinase1
GABARAP: Gamma-aminobutyric acid receptor-associated protein
MDVs: mitochondrial-de rived vesicles
mtDNA: mitochondrial DNA
ETC: electron transport chain
tRNA: transfer RNA
rRNA: ribosomal RNA
ATP: adenosine triphosphate
DAMPs: Damage-associated molecular patterns
TNF-α: tumor necrosis factor α
IFN-γ: interferon-γ
SASP: senescence-associated secretory phenotype
DRP 1: dynamin-related protein 1
HIF-1 α: hypoxia-inducible factor 1-alpha
mtROS: mitochondrial reactiv e oxygen species
CIA: collagen-induced arthritis
Trm: tissue-resident memory T
Th1: type 1 helper T cell
Th17: T helper cell 17
IL-17A: interleukin-17A
mTORC1: mechanistic target of rapamycin co mplex 1
RAPA: rapamycin
AIA: antigen-induced arthritis
ASIC1a: acid-sensing ion channel 1a
Ca N: calcineurin
HSP70: heat shock protein 70
SIRT3: sirtuin 3
LAMP1: lysosome-associated membrane protein 1
NLRP3: nucleotide-binding oligomerization d omain, leucine-rich repeat and pyrin domain- containing 3
FLS: fibroblast-like synoviocytes
IL-1 β: interleukin-1β
IL-18: interleukin-18
RA-FLS: fibroblast-like synoviocytes
H₂O₂: hydrogen peroxid e
AA: adjuvant arthritis
KEAP1: Kelch-like ECH-associated protein 1
Nrf2: nuclear factor-erythroid 2 related factor 2
ANGPTL2: angiopoietin-lik e 2
IGFBP5: insulin-like growth factor-binding protein 5
MiD49 and MiD51: MiDs, mitochondrial dynamics proteins of 49 and 51 kDa
CPT-1A: carnitine palmitoyltransferase 1A
MAPK: mitogen-activ ated protein kinase
STAT3: signal transducer and activator of transcription 3
TREM1: triggering rec eptor expressed on myeloid cells 1
TOMM40: translocase of outer mitochondrial membrane 40
E2F1: E2F transcription factor 1
APJ: Apelin receptor
AMPK: AMP-activated protein kinase
GSZD: Guizhi-Shaoyao-Zhimu Decoction
GSZG: Guizhi-Shaoyao-Zhimu granules
NF-κB: nuclear factor kappa-B
US: Ultrasound
NSs: two-dimensional pi ezoelectric nanosheets
